# External validation of a collar-mounted triaxial accelerometer for second-by-second monitoring of eight behavioural states in dogs

**DOI:** 10.1371/journal.pone.0188481

**Published:** 2017-11-29

**Authors:** Ingrid den Uijl, Constanza B. Gómez Álvarez, David Bartram, Yoni Dror, Robert Holland, Alasdair Cook

**Affiliations:** 1 vHive, School of Veterinary Medicine, University of Surrey, Guildford, United Kingdom; 2 Zoetis International Office, Dublin, Ireland; 3 Oggii Ltd, Tel Aviv, Israel; University of Sydney Faculty of Veterinary Science, AUSTRALIA

## Abstract

Early detection of disease by an animal owner may motivate them to seek early veterinary advice. Presentation before a more advanced clinical manifestation is evident could lead to more effective treatment and thus benefit the animal’s health and welfare. Accelerometers are able to detect changes in specific activities or behaviours, thus indicating early signs of possible adverse health events. The objective of this validation study was to determine whether the detection of eight behavioural states: walk, trot, canter/gallop, sleep, static/inactive, eat, drink, and headshake, by an accelerometer device was sufficiently accurate to be useful in a clinical setting. This fully independent external validation estimated the accuracy of a specific triaxial, collar-mounted accelerometer on a second-by second basis in 51 healthy dogs of different breeds, aged between 6 months and 13 years, weighing >10 kg. The overall diagnostic effectiveness was estimated as: % record correctly classified of > 95% in walk, trot, canter/gallop, eat, drink and headshake and >90% in sleep and static/inactive. The positive predictive values ranged from 93–100%, while the negative predictive values ranged from 96–100%, with exception of static/inactive (86%).This was probably because dogs were placed in unfamiliar kennels where they did not exhibit their typical resting behaviour. The device is worn on a collar, making its use feasible for anyone wanting to monitor their dog’s behaviour. The high accuracy in detecting various kinds of behaviour appears promising in assessing canine health and welfare states.

## Introduction

The use of digital technology in veterinary medicine has been increasing rapidly and will keep growing with the current increase of innovations in this field. Telemedicine will become an integral part of the practice of certain aspects of veterinary medicine as it has in human medicine [1]. With advancing connectivity, data derived from wearables or other sensors promises to enable digital disease surveillance [2]. Accelerometers have been used in humans for gait analysis [3–6] as well as study of circadian rhythms [7, 8], prediction of adverse health events [9] and energy expenditure [10]. Accelerometers, also called inertial sensors, have been used in horses for decades to detect and monitor lameness [11–14]. These sensors have also been used in horses for lying behaviour [15] and in wild animals for tracking behaviour [16]. In dogs, accelerometers have been used to monitor amount of activity [17–21], activity types [22], cognitive dysfunction [23, 24] and for lameness detection [21, 25].

Common clinical conditions of dogs that could be detected by an accelerometer include diabetes [26], dermatologic and musculoskeletal problems [27], ear infections [28], and mental health conditions, such as separation distress [29, 30]. In clinical practice, under- and late reporting of changes in health are commonly recognized [10, 31, 32] which have the potential to delay clinical interventions. Currently, presentation of a clinical case to a veterinary practice is dependent on the owner’s ability to detect the onset of disease and their attitude towards the value of veterinary advice. Early detection of disease by the owner may enable them to present their dog to their veterinarian in an early stage of the disease, which could lead to more effective treatment and thus benefit the animal’s health and welfare. Accelerometers are able to detect changes in specific activities or behaviours, indicating signs linked to possible adverse health events [24, 25]. Triaxial accelerometers can detect behavioural states by measuring accelerations in three axes, which can be analysed resulting in description of motion in different planes. The data can then be analysed by bespoke algorithms applied to data from specific accelerometer devices to automatically predict such states.

In order for an accelerometer to be adopted by the general public and veterinary practices the device has to be small, lightweight, energy efficient and cheap [22] as well as shock- and waterproof for outdoor use. Many apps, wearables or other innovative devices are adopted by customers but are abandoned due to disinterest and disenchantment. To promote continued compliance, it is firstly necessary that any device addresses an issue that is important to the user. In addition, it must be easy to use and deliver a regular output that is easy to interpret by the user [1]. In the future, big data that are derived from wearables will enable veterinarians to measure health and welfare outcomes objectively for digital disease surveillance, remote post-operative care or treatment efficacy. Since data, once aggregated, can be re-used for multiple purposes, these information assets can be utilised for business, research and educational purposes.

After performing an internal validation study [33], the objective of this external and independent validation study was to show that the detection of behavioural states by an accelerometer device is sufficiently accurate to be useful in a clinical setting. This is a necessary prerequisite to investigating the role of such devices to predict a change in health status of an individual dog. The study investigated the accuracy of a specific triaxial, collar-mounted accelerometer on a second-by second basis in healthy dogs for the detection of eight behavioural states: walk, trot, canter/gallop, sleep, static/inactive, eat, drink, and headshake.

## Material and methods

### Device

The triaxial, collar-worn accelerometer device (PetDialog+ powered by Oggii, Zoetis) detects eight behavioural states of a dog. It incorporates a battery with a life of at least a year, after which a new device can be acquired and the data can be transferred to ensure longitudinal collection of data. The device gathers data and sends it to a central database by a Bluetooth application. Data are processed by algorithms developed by Oggii Ltd, Tel Aviv, Israel, where the behavioural state displayed by a dog is determined each second. The time spent in each behavioural state is displayed in the owner’s smart phone app on a daily basis (PetDialog, Zoetis), a longitudinal display of the activity data will be available for veterinarians to interpret via an online dashboard (VetSupport+, Zoetis).

### External validation

The manufacturer performed a split-sample internal validation, with short instances of well-defined behaviour [33]. This differs from the way that owners and veterinarians would use the device, as they would use continuously recording of current and subsequent behaviours. Therefore, to complete the validation process a fully independent external validation was performed to support the general applicability of the outcome of the algorithms [34] by addressing geographical, methodological and spectrum transportability [35].

In this prospective study the device was tested in a manner similar to that in which it will be used by dog owners. Participating dogs were fitted with the device on their collars and taken for a walk on and off leash on the same pre-determined track at the grounds of the University of Surrey. The track comprised a combination of pavements, grass and gravel paths. A video recording of each dog was made and synchronised with the data derived from the device. The off leash behaviour was filmed in a large contained area on a grass field, fenced with sheep fencing.

### Study population

We asked the general public in Surrey, United Kingdom to volunteer with their dogs for the study via flyers posted on message boards in dog walking areas. Eligibility criteria were that dogs should be healthy, with a bodyweight of ≥10 kg and of any breed. The algorithms were trained for dogs of 10 kg and over, because although the device is not heavy, it is rather large to put on a small dog’s collar. Prior to participation the dogs were clinically examined by a veterinarian and a behavioural assessment (ASPCA SAFER®) was completed by appropriately trained staff. In total, 53 dogs were volunteered, but two dogs had to be excluded due to lameness found at the clinical examination prior to participation. Data from 51 dogs were collected from May-October 2016 at suitable times for the owners.

### Filming protocol

**Fig 1:**
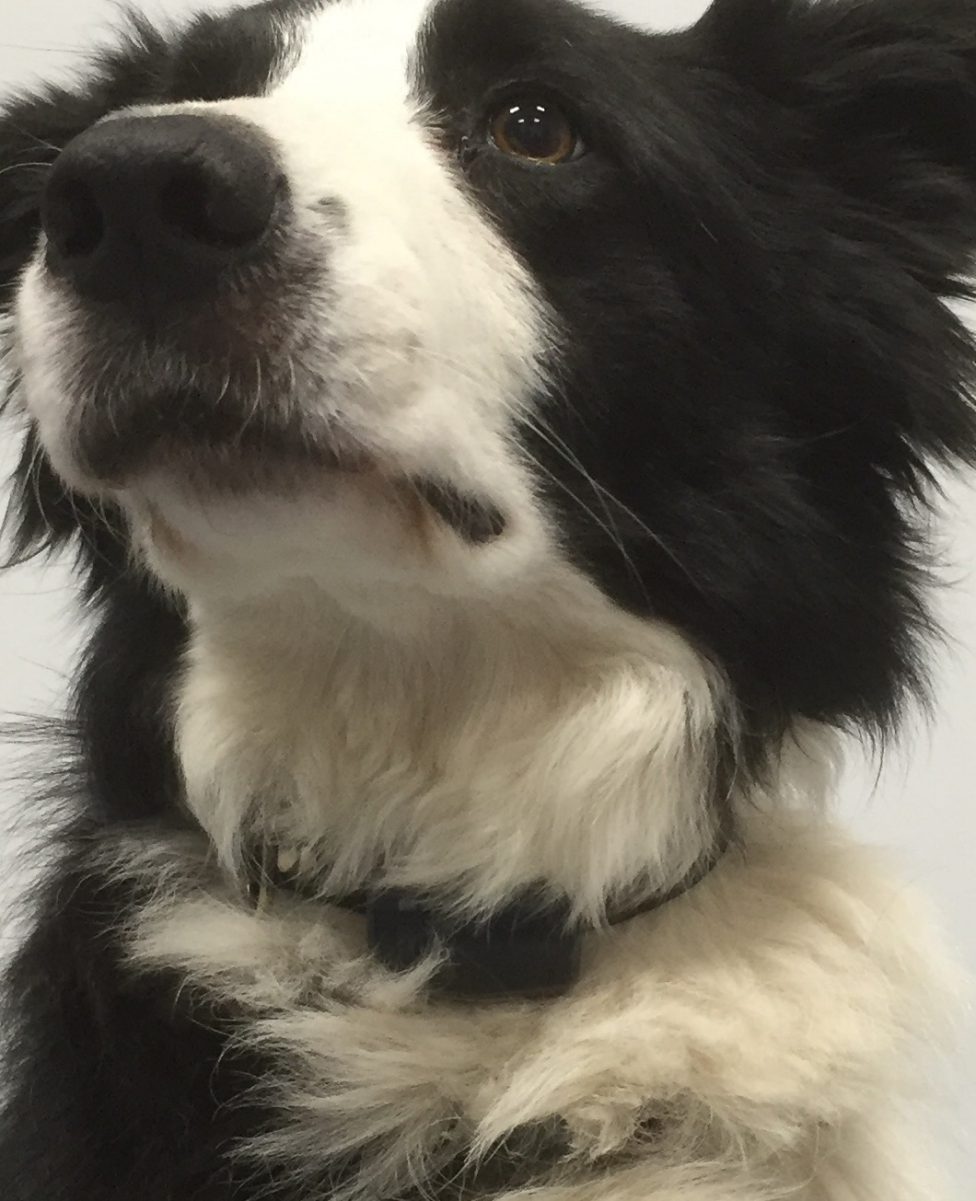
Dog wearing the triaxial accelerometer on its collar.

Participating dogs were fitted with a device, on the ventral aspect of their collar, according to the device instructions. The collar was adjusted to fit the dog’s neck comfortably (Fig 1). The dogs were filmed whilst wearing the device with a hand held HD camcorder (Panasonic HC-V250) using a standardised protocol. Dogs were filmed so that the camera captured their side view at all times. At the beginning of each session a protocol was initiated to accurately synchronise the video and the device. It was important to avoid offsets of more than 1 second which would have created misalignments between algorithm results and recorded observation results. First the electronic clock screen was filmed, then the person leading each dog flipped the device from side to side twice with an approximate 30 second interval.

During the session, dogs were encouraged to perform each activity type at least twice for a period of homogenous activity, with exception of headshaking, eating and drinking which were allowed to be shorter. Dogs and their study handler were able to freely choose the sequence and pace of the activities and the study period was filmed continuously. At the beginning of each session dogs were let off leash for 10–15 minutes in order to observe and record off leash activity (static, walk, trot, canter/gallop). The end of each session was indoors, sampling eat, drink, and sleep if possible. During eating and drinking, the bowl content was clearly visible in the video.

### Tagging

The analyses of the video recordings were used as the gold standard. First, the data from the device and the film were synchronised using custom-made software (Oggway Ltd, Tel Aviv, Israel). Then the video recordings were classified (tagged) according to a predetermined standardised observational guideline (S1 Text) by three specially trained taggers. Each sequence of behaviour was allocated a behavioural state as observed by the tagger. Taggers could choose from any of the eight behavioural states and would tag the beginning and end of the behavioural sequence. The taggers did not have access to the states predicted by the algorithm. Time frames of more than 3 seconds of continuous activity which were clearly defined from the video were tagged, with exception of headshakes. Due to their short duration (1–2 seconds), headshakes were registered per event.

Data were not tagged in the following circumstances: if the dog was not clearly visible in the video; the dog was pulling on the lead; the dog was petted or touched by a person; someone was blocking the view, or if the dog showed combined behaviour (e.g. jumping while trotting etc.). Any changes within 2 sec were also not tagged, with exception of headshaking and lifting their head while eating or drinking. The video data was tagged as duration of state, with a start and end time, using custom-built software for tagging of the different activity types (Matlab®, MathWorks, UK).

### Classification of behaviour

The current study shows the validation of eight behavioural states predicted by the analytical algorithms that were created by the developers. These algorithms were trained using various behavioural states from 3000 individual dogs. The algorithm is hierarchical, it first predicts headshakes and subsequently classifies all other behavioural states. Table 1 shows the definition of the eight behavioural states.

**Table 1:** Description of eight behavioural states predicted based on data collected from the triaxial accelerometer.

Behavioural state	Description
**Walk**	A slow pace, by advancing the feet alternately so that there are always two or more feet on the ground
**Trot**	A moderately fast, two-beat and symmetric gait, includes pacing
**Canter/gallop**	The fastest asymmetric canine gait, includes canter and gallop
**Static/inactive**	The dog changes postures without actively change his/her location, includes sit, lie down (with head up) and standing
**Sleep**	Continuous state of inactivity, dog lies in relaxed posture with head on the floor
**Eat**	Dog eats food from a bowl (head lowered to bowl), head lifting and looking around, steps in place are excluded from this state
**Drink**	Dog drinks water from a bowl (head lowered to bowl), head lifting and looking around, any movement with raised head in between sips are excluded from this state
**Headshake**	The dog’s head turns left and right and/or rotates so that the ears move up and down repeatedly. One shake consists of the start of the movement until the head is static again.

After completion of filming the data from the device was immediately uploaded via Bluetooth and the behavioural states were determined by the algorithm and each time the dog changed state, ID, time and state were stored in log files (.osl).

### Ethics statement

The study protocol was submitted to the Non-Animal Scientific Procedures Act sub-committee of the Animal Welfare and Ethical Review Body of the University of Surrey (Permit Number NASPA-2016-003-SVM), which provided a favourable ethical opinion that satisfied the requirements of the University’s Research Integrity and Governance Office. This constituted approval for the study. Participants signed written informed consent prior to the start of their study protocol. All efforts were made to minimise stress and ensure the welfare infringements of the participating dogs in accordance with the University’s ethical conduct policy. No coercion was used if any dog did not display any behaviour naturally.

### Sample size calculation

A sample size calculation was performed, based on the data collected for testing the device by the manufacturer (S1 Table). The expected sensitivities of the eight behavioural activities was 0.85–0.95. The expected specificities of the eight behavioural activities was 0.96–1.0 [33]. Based on these figures the sample size for the sensitivity of 0.85 (sleep) will generate the most conservative estimate of sample size. If there were sufficient dogs to validate sleep, then all eight activities can be validated. To validate a sensitivity of 0.85 with 95% confidence and 10% precision required 48 dogs. This number was calculated by using the single proportion sample size formula to calculate the number of dogs needed for true positive and false negative. The natural occurrence of the behaviour was then used to determine the definitive sample size [36].

### Data analysis

Both the file with the states as predicted from the data collected by the device and the file with the tagged data were transformed into second by second files and merged on ID and time. If a behaviour ended in the same second as the next behaviour started, the behaviour of the start time was taken and the last second of the previous behaviour was discarded.

Headshakes were counted as number of headshakes, rather than seconds. Any headshakes within a 5 second window were counted as one headshake. For all other states only behaviours ≥4sec were included in the analysis as anything less is visually difficult to detect, with exception of eating and drinking, which were included ≥2sec.Transitions are short periods of time where a dog changes from one behaviour into another. During these periods, for example while changing gait, the dog does not show a clear behaviour. Therefore the first and the last second of each state were excluded from analysis.

### Diagnostic accuracy

The data were analysed to determine agreement between the tagged behaviour (as observed on video) and the classification determined each second by the algorithms based on the triaxial accelerometer device.

The sensitivity, specificity, positive predictive value (PPV) and negative predictive value (NPV) of the states were analysed and diagnostic accuracy was determined by the positive and negative likelihood ratio and the diagnostic effectiveness [37]. Diagnostic effectiveness is dependent on the prevalence, while likelihoods are not and can therefore be compared across populations [38]. A test with a positive likelihood ratio (LR+) >10 and a negative likelihood ratio (LR-) <0.1 was considered a good test.

Data were analysed on a second-by-second basis, each second is therefore regarded as a single measurement. To account for multiple measurements per dog, parameter values were first calculated per dog and activity type and then aggregated by activity type, weighted for the frequency of contribution per dog, quoting 95% confidence intervals.

All data management and statistical analyses were performed using StataSE14, Statacorp, TX, USA.

## Results

### Study population

The characteristics of the study population for the internal validation and external validation are presented in Table 2. All selected dogs were healthy while participating in the study. The study comprised 51 dogs from 36 breeds or crossbreeds that were classified in nine categories. Mean weight was 25kg (range 10-42kg), and mean age was 4.6 year, ranging from 6 months to 13 years. The majority of dogs were between 1 and 8 years old. Twenty-three were female (45%), and 28 dogs were male (55%). Almost all dogs were neutered or spayed (82%), no significant difference was found between neutered male and female dogs (p = 0.15).

**Table 2:** Characteristics of study population (n = 51 dogs).

		No. (percentage)
**Breed Category**		
	Retriever	12 (24%)
	Shepherd	11 (22%)
	Spaniel	7 (14%)
	Hunting dog	6 (12%)
	Collie	6 (12%)
	Unknown/Other	4 (7.8%)
	(Bull) terrier	3 (6.0%)
	Sled dog	1 (2%)
	Small dog	1 (2%)
**Weight**		
	10–15 kg	9 (18%)
	15–20 kg	10 (20%)
	20–25 kg	7 (14%)
	25–35 kg	18 (35%)
	≥ 35 kg	7 (14%)
**Age**		
	0–1 year	2 (4.0%)
	1–3 years	15 (30%)
	3–5 years	14 (28%)
	5–8 years	13 (26%)
	≥ 8 years	6 (12%)
**Gender**		
	Female	23 (45%)
	Male	28 (55%)
**Intact**		
	No	42 (82%)
	Yes	9 (18%)

### Diagnostic accuracy

Table 3 presents an overview of the diagnostic parameters for each state. Sensitivity varies from 0.86 (static/inactive) to 0.98 (headshake), specificity ranges from 0.66 (sleep) to 0.97 (static/inactive). For owners the predictive values are very important as these show that the behaviour predicted by the device is that which the dog has displayed. The positive predictive values, the probability that the device states the behavioural state in accordance with the behavior on video, varied from 0.93 (sleep) to 1.00 (trot). Negative predictive value, the probability that the device does not identify a behaviour when the dog is not showing it in the video, was also high, ranging from 0.98 to 1.00, with exception of sleep (NPV = 0.85).

**Table 3:** Sensitivity, specificity, PPV^a^ and NPV^b^ for each state.

State	N	Sensitivity (95% CI)	PPV (95% CI)	Specificity (95% CI)	NPV (95% CI)
**Walk**	48	0.91 (0.86–0.96)	0.99 (0.98–0.99)	0.91 (0.86–0.95)	0.98 (0.97–0.99)
**Trot**	46	0.91 (0.88–0.95)	0.98 (0.97–0.99)	0.78 (0.72–0.85)	0.99 (0.99–1.00)
**Canter/gallop**	43	0.96 (0.92–1.00)	1.00 (1.00–1.00)	0.92 (0.88–0.96)	1.00 (1.00–1.00)
**Sleep**	27	0.95 (0.87–1.00)	0.93 (0.89–0.97)	0.66 (0.52–0.79)	1.00 (1.00–1.00)
**Static/inactive**	51	0.86 (0.81–0.90)	0.98 (0.96–0.99)	0.97 (0.96–0.99)	0.85 (0.79–0.91)
**Eat**	23	0.92 (0.83–1.00)	0.99 (0.98–1.00)	0.73 (0.59–0.87)	1.00 (1.00–1.00)
**Drink**	23	0.89 (0.74–1.00)	1.00 (1.00–1.00)	0.87 (0.72–1.00)	1.00 (1.00–1.00)
**Headshake**	51	0.98 (0.96–1.00)	0.99 (0.99–1.00)	0.95 (0.92–0.98)	0.99 (0.99–1.00)

^a^ Positive predictive value.

^b^ Negative predictive value.

Table 4 presents the diagnostic effectiveness and likelihood ratios of each state. The diagnostic effectiveness, the proportion of correctly classified records was over 95% for all states, except static/inactive (91%) and sleep (94%). The likelihoods show that the predictions made by the device are good, only the LR-, for static/inactive and drink are higher than 0.1 showing that drink and static may be reported by the device when on video dogs were displaying other behaviours.

**Table 4:** Diagnostic accuracy for each state.

State	Diagnostic effectiveness	LR+ ^a^	LR- ^b^
**Walk**	0.98 (0.97–0.99)	162 (94–230)	0.09 (0.04–0.14)
**Trot**	0.98 (0.97–0.99)	195 (91–299)	0.09 (0.05–0.13)
**Canter/gallop**	1.00 (1.00–1.00)	559 (281–838)	0.04 (-0.01–0.08)
**Sleep**	0.94 (0.91–0.98)	31 (9.4–52)	0.06 (-0.01–0.13)
**Static/inactive**	0.91 (0.87–0.94)	94 (61–126)	0.16 (0.10–0.22)
**Eat**	0.99 (0.98–1.00)	245 (83–408)	0.08 (-0.02–0.17)
**Drink**	1.00 (1.00–1.00)	1059 (328–1790)	0.11 (-0.04-.27)
**Headshake**	0.95 (0.94–0.97)	98 (96–100)	0.02 (-0.01-.04)

^a^ Positive likelihood.

^b^ Negative likelihood

After analysis of the data the footage of almost all of the discrepancies between the prediction of the algorithm and the tagged data were reviewed. The confusion matrix shows the raw outcome of the algorithm unadjusted for multiple measurements per dog. It shows which behaviours were classified similarly between taggers and the device and where disagreement was detected. (S2 Table). It shows that in almost half of the frames where the device registered sleep, the behaviour on video was tagged as static/inactive. On review of the footage, it was noticed that the dogs that were classified as static were lying down in the kennels, but with their heads up and extremely still. These dogs were observed to be alert despite their recumbent position.

Three dogs (all collie breeds) showed pacing. Based on speed and movement pattern (i.e. not lifting all limbs from the floor), walk was tagged in these dogs, while the device classified it as trot, because it was detected as a two beat gait. On review of the footage at slowed down speed (10% of normal speed), pacing was detected visually in these dogs.

Fewer than half of the participating dogs ate or drank during the validation study. Owners were asked to bring the dog’s own food, unfortunately some owners had forgotten food, some dogs had already been fed in the morning and were not hungry anymore, while others simply did not want to eat in an unfamiliar environment. The dogs had ad lib access to water and tended to take little sips rather than long drinks. In between sips and bites dogs were looking around at their environment, which the algorithm confused eating and drinking with static/inactive if the sip or nibble was short in duration compared to the looking around time (S2 Table).

## Discussion

To the best of our knowledge, this is the largest external validation study of a device designed to detect eight different behavioural states in dogs. The detailed detection of the behaviour of dogs allows the device to be used for monitoring of health in clinical practice. The accuracy of this device was excellent in all eight behavioural states, except with sleep and static/inactive behavioral states, which performed slightly less accurately than other states. Dogs were observed to be alert despite their recumbent position in kennels and were tagged static/inactive, however the device classified this behaviour as sleep. An increased period of sleeping behaviour should be carefully evaluated in case the dog remains static/inactive for too long instead of sleeping, which could indicate some other issues such as anxiety and hypervigilance. When monitoring canine health with the app and the device, dogs will be compared to their individual baseline and changes in that baseline will trigger alerts to be investigated by veterinarians. During this study some dogs that were pacing were classified as trotting, because both are two-beat gaits. It is expected that this classification will not affect the detection of change in activity pattern, as the increase of pacing will be reported as an increase in trotting. However, pacing can only be identified visually during clinical examination and not with the device.

The device sometimes labelled drinking and eating as static/inactive, because of the short moments of the actual behaviour in between looking up from the bowl. A possible solution to this would be to change the algorithm to not only predict the second-by-second behaviour, but also take into account the behavioural states within a certain time period before the actual prediction. So instead of predicting a behavioural state, it would predict an activity, which may include several states in a predetermined pattern. For example for eating the patterns could include having a bit, looking around and eating some more. Almost half of the study population showed eating and drinking behaviour, a lower number than anticipated in the sample size calculation, therefore the confidence intervals are a little larger for eating and drinking than the other states. These states would benefit from further validation in a home environment.

We used human observation as gold standard. In long sequences it was assumed that the differences between the device and tagged data were due to the same error. Generally, after slowing down the footage significantly, these reviewed sequences demonstrated that the device correctly predicted the exhibited behaviour and the observer had been mistaken. For example, dogs can move quickly from one behavioural state to another, sometimes not visible for the human eye, e.g. three paces of walk in a stretch of trotting was picked up by the device, but had to be played at almost 25% of real time for taggers to be able to identify the brief walk. To the human eye it looked like one continuous trot, while in most cases on review the device was rightly classifying different behaviour.

Compared to other accelerometer studies, the accelerometer in the current study was capable of detecting more behavioural states than any other published accelerometer. In particular, eating and drinking as well as headshaking, important to detect itching etc. has not been investigated previously. Michel et al, 2009, studied an accelerometer which could distinguish walking from trotting and sedentary dogs. They found that they could perfectly distinguish walking from sedentary and found sensitivities and specificities for walking and trotting comparable to those in the current study [20]. Compared to Gerensér et al, 2013, the current study comprised twice as many subjects. That study was an internal validation of a triaxial accelerometer combined with a gyroscope, which allowed them to investigate seven gait categories; lay, sit, stand, walk, trot, gallop and canter. The device was mounted on a custom made harness and showed similar results when the training and validation were performed on datasets comprising the same dogs, but had significantly less success when the algorithm was validated on a training set with different dogs [22]. That study emphasises the importance of independent external validation, as in the current study, where algorithms that were trained on one population of dogs were validated with an unrelated population. Contrary to a commonly used internal or cross-validation, an external validation has the advantage of being independent and unbiased [34]. Although an opportunistic sample was used and participating dogs were not selected at random, the recruited dogs presented differing characteristics (e.g. age and breed distribution), addressing spectrum transportability. Additionally, they were tested at a different geo-location than the original population on which the algorithm was trained and the internal validation was performed, addressing geographical transportability. Moreover, dogs were filmed continuously whilst performing natural behaviour during a walk, methodological transportability, which was comparable to the use of the device in practice. Commonly, the validation of accelerometers is conducted in laboratory-based experiments using a treadmill, which can alter the animals gait, or by filming trained animals [16]. As with Michel et al, 2009 [20], the device was placed on the ventral collar, which has been reported as most convenient location, with an acceptable correlation with videography imaging compared to a different location on the body [19]. Placement on the collar allows the device to be easily used and cheaper in practice compared to a specially designed harness that needs to be purchased with the device as in the study of Gerensér et al, 2013 [22].

This study demonstrates that the accuracy of this device is sufficient for use in a clinical setting. The next step is to look at the circadian rhythms of individual dogs and develop a baseline that takes into account owner habits and environmental circumstances, like daylight savings, weather etc. Subsequently, alerts may be developed to highlight potential health threats in an early stage, possibly before serious clinical signs develop. For example, any decrease in specific gait patterns (walk, trot, canter/gallop) could be an indication of cardiac disease or osteoarthritis [39] or hypothyroidism [23], especially when combined with other changes in other states like eating or drinking. An increase in shaking activity could be a sign of dermatologic conditions such as otitis or pruritus [39]. Changes seen in the amount of food and water intake patterns could account for many different conditions, for example, a loss in appetite could be a result of liver disease. However, an increase of appetite may be caused by diabetes mellitus, pancreatic disease or internal parasites and an increase in drinking could be a sign of diabetes insipidus or kidney disease. Additionally, an increase in shake activity could be a sign of ear mites or otitis [28], or could be similar to head tremors, a sign of neurological disease [40]. Establishing a dog’s average daily circadian rhythms and detailed records of different dog behaviours, will provide researchers, veterinarians and pet owners an unprecedented understanding of dog’s daily habits, even in the absence of humans, and opportunities to objectively investigate health and welfare in the dog population.

## Conclusions

The triaxial accelerometer studied, has a high diagnostic accuracy as shown by high sensitivity, specificity, PPV and NPV. Diagnostic effectiveness, the proportion of records correctly classified, was very high, >95% for walk, trot, canter/gallop, eat, drink and headshake and >90% for static/inactive and sleep. The positive predictive values were all >93%, meaning that when the device classified a certain behaviour, the dog was usually displaying it. The device can be worn on a collar, making it feasible for anyone wanting to monitor their dog’s behaviour. The high accuracy of the device in detecting various kinds of behaviour appears promising in assessing canine health and welfare states.

## Supporting information

S1 Text Description of behavioural states. (DOCX)

S1 Table Sensitivity and specificity per state from the internal validation. (DOCX)

S2 Table Confusion matrix of agreement of frames. (DOCX)
