# Supplementary material for: External validation of a collar-mounted triaxial accelerometer for second-by-second monitoring of eight behavioural states in dogs
Source: PLoS One. 2017 Nov 29;12(11):e0188481. doi: 10.1371/journal.pone.0188481 (PMC5706712; doi:10.1371/journal.pone.0188481)
Supplement: S1 Text — (DOCX) [file pone.0188481.s001.docx]

**S1 Appendix. Description of behavioural states**

1. **Walk (gait)**
   1. Definition: Advancement on foot at a moderate speed or pace; proceed in steps; move by advancing the feet alternately so that there is always two or more feet on the ground. For example: left front leg, right hind leg, right front leg, left hind leg in a regular 1-2-3-4 beat. (Amble in the diagram).
   2. Description: The dog progresses forward; typical walking speed and pattern; Head movements should be included in this activity.
   3. Emphasis: Other activities in between “walk” such as stopping, running etc., should be excluded and registered per other activities definitions.
2. **Trot (gait)**
   1. Definition: A moderately fast, two-beat and symmetric gait.
      1. Trot in which the legs move in diagonal pairs.
      2. Pace in which front and hind legs of the same side move in parallel.
   2. Description: The dog progresses forward; typical symmetric running speed and pattern; Head movements will be included in this activity.
   3. Emphasis: Other activities in between “run” such as stopping, walking etc., should be excluded and registered per other activities definitions.
3. **Canter/gallop (gait)**
   1. Definition: The fastest asymmetric canine gait.
      1. Canter three-beat fast gait.
      2. Gallop four-beat fast gait.
   2. Description: The dog progresses forward; typical asymmetric sprint speed and pattern.
   3. Emphasis: Other activities in between “sprint” such as stopping, running, walking etc., should be excluded and registered per other activities definitions.
4. **Sleep**
   1. Definition: No body movements for at least 5 continuous minutes.
   2. Description: The dog lies on the floor / other surface. His/her body is totally relaxed.
   3. Emphasis: Any activity in between “sleep” activity (changing position, head lifting etc.) should be excluded and registered per other activities definitions.
5. **Static**
   1. Definition: The dog changes postures without actively change his/her location.
   2. Description: the dog stands/sits/lies/changes positions.
   3. Emphasis: a few steps (2-3), **not strides**, within the same location should be considered as “static”.
6. **Eat**
   1. Definition: The dog eats from his/her bowl while standing above it.
   2. Description: The dog picks objects from the bowl into his/her mouth, chews, and swallows it.
   3. Emphasis:
      1. Slight head lifting while chewing should be considered as “eat”.
      2. Other activities such as full head lifting and looking around, steps in place etc. should be excluded and registered per other activities definitions.
7. **Drink**
   1. Definition: The dog drinks from his/her bowl while standing above it.
   2. Description: The dog picks liquid into his mouth and swallows.
   3. Emphasis:
      1. Other activities in between “drink” such as head lifting and looking around, steps in place etc. should be excluded and registered per other activities definitions.
8. **Headshake**
   1. Definition: The dog’s head turns left and right or rotated so the ears move up and down repeatedly. One shake consists of the start of the movement until the head is static again.
   2. Emphasis: shake duration is ~1-2 seconds.
