# Supplementary material for: External validation of a collar-mounted triaxial accelerometer for second-by-second monitoring of eight behavioural states in dogs
Source: PLoS One. 2017 Nov 29;12(11):e0188481. doi: 10.1371/journal.pone.0188481 (PMC5706712; doi:10.1371/journal.pone.0188481)
Supplement: S1 Table — (DOCX) [file pone.0188481.s002.docx]

**Supplement 2 Table A. Sensitivity and specificity from the internal validation**

|  | **Sensitivity** | **Specificity** |
| --- | --- | --- |
| **Walk** | 0.92 (0.91-0.92) | 0.98 (0.98-0.98) |
| **Trot** | 0.95 (0.95-0.96) | 0.99 (0.99-0.99) |
| **Canter/gallop** | 0.95 (0.94-0.96) | 1.00 (1.00-1.00) |
| **Sleep** | 0.85 (0.85-0.86) | 0.99 (0.99-0.99) |
| **Static/inactive** | 0.94 (0.93-0.94) | 0.97 (0.97-0.97) |
| **Eat** | 0.85 (0.84-0.86) | 0.96 (0.96-0.97) |
| **Drink** | 0.74 (0.73-0.75) | 0.98 (0.98-0.99) |
| **Headshake** | 0.92 (0.90-0.93) | 1.00 (1.00-1.00) |

These values are adjusted for repeated measurements per dog.

Adapted from: den Uijl I G-AC, Dror Y, Manning N, Bartram D, Cook A. Validation of a collar-mounted accelerometer that identifies eight canine behavioural states, including those with dermatologic significance. Proceedings of British Veterinary Dermatology Study Group; 2016; p81-84.
