# Supplementary material for: External validation of a collar-mounted triaxial accelerometer for second-by-second monitoring of eight behavioural states in dogs
Source: PLoS One. 2017 Nov 29;12(11):e0188481. doi: 10.1371/journal.pone.0188481 (PMC5706712; doi:10.1371/journal.pone.0188481)
Supplement: S2 Table — (DOCX) [file pone.0188481.s003.docx]

**Supplement 3 Table B. Confusion matrix of agreement of frames**

|  |  | **Video (taggers)** | | | | | | |
| --- | --- | --- | --- | --- | --- | --- | --- | --- |
|  |  | **Walk** | **Trot** | **Canter/**  **Gallop** | **Sleep** | **Static/ Inactive** | **Eat** | **Drink** |
| **Triaxial accelerometer (device)** | **Walk** | *9,832* | 148 | 1 | 2 | 182 | 59 | 7 |
|  | **Trot** | 560 | *2,238* | 15 | 0 | 15 | 53 | 0 |
|  | **Canter/gallop** | 20 | 83 | *750* | 0 | 2 | 0 | 0 |
|  | **Sleep** | 0 | 0 | 0 | *6,536* | 5,861 | 64 | 0 |
|  | **Static/inactive** | 104 | 13 | 15 | 226 | *19,816* | 13 | 3 |
|  | **Eat** | 92 | 0 | 1 | 0 | 619 | *873* | 15 |
|  | **Drink** | 60 | 0 | 0 | 15 | 52 | 53 | *116* |

These values are unadjusted for repeated measurements per dog. Each frame represents one second of behavior.

The confusion matrix shows the raw outcome of the algorithm u adjusted for multiple measurements per dog. It shows which behaviours were classified similar between taggers and the device and what the disagreement between the two was.
